# Correlation analysis of microstructure, protein pattern, and thermal properties of *Procambarus clarkia* subjected to different cryogenic treatments

**DOI:** 10.1002/fsn3.2445

**Published:** 2021-07-06

**Authors:** Liu Shi, Xin Li, Guangquan Xiong, Wenjin Wu, Anzi Ding, Yu Qiao, Li Liao, Dongqing Chen, Lan Wang

**Affiliations:** ^1^ Key Laboratory of Refrigeration and Conditioning Aquatic Products Processing Ministry of Agriculture and Rural Affairs Xiamen China; ^2^ Institute of Agricultural Products Processing and Nuclear‐ agricultural Technology Hubei Academy of Agricultural Sciences / Farm Products Processing Research Sub‐center of Hubei Innovation Center of Agriculture Science and Technology Wuhan China; ^3^ Fujian Anjoy Food Co Ltd, Xiamen China

**Keywords:** correlation analysis, microstructure, protein pattern, red swamp crayfish, thermal properties

## Abstract

The objective of this work was to investigate the freezing and storage temperature (−80 and −18℃) on the microstructure, protein pattern, and thermal properties of red swamp crayfish after one‐week storage, and a Pearson correlation analysis was performed among these attributes. After cryogenic treatments for short‐term storage, Tp (pretein denaturation temperature) was significantly raised (*p* < .05) except for samples frozen at −80℃ prior to store at −18℃ (−80/‐18). Samples frozen and stored at −80℃ (−80/‐80) had lower number and sum area of white regions in histology, higher intensity of most protein bands in sodium dodecyl sulfate polyacrylamide gel electrophoresis (SDS‐PAGE) image, and relatively higher Tp and ΔH (*p* < .05), while −80/‐18 samples had lower intensity of most protein bands and TP 2, and higher number and sum area of white regions and ΔH 2 (*p* < .05). Pearson's analysis results showed the intensive TN T and MLC 1 band could be potentially considered as the markers of tissue integrity and protein degradation. Therefore, the three attributes could be applied to comprehensively assess the quality of frozen aquatic products, and −80/‐80 treatment was appropriate for crayfish preservation.

## Graphical abstract

TN T and MLC 1 band revealed crayfish tissue integrity and protein degradation.

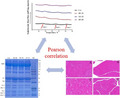

## 1 INTRODUCTION

Red swamp crayfish (*Procambarus* *clarkia)*, well known for its special flavor, has become the most important freshwater fishery resource in China. The cultivation amount and the annual economic output of red swamp crayfish in 2018 were 1.64 million ton and 369 billion RMB, respectively (Anonym, 2019). Except sold alive in local market during April to September, frozen flavored crayfish product, as its convenience and tasty, is becoming more and more popular for young families and considered as rinsing star product in E‐commerce.

Freezing is an effective technique to preserve food, especially in food industries. Low temperature could effectively inhibit the reproduction of microbial and retard the quality deterioration of food products (Leygonie et al., 2012). Several works have been done to investigate the effects of freezing on the meat quality during frozen storage (Choi et al., 2017a, 2017b; Dalvi‐Isfahan et al., 2016; Shi, Tao, et al., 2018). Beside texture, microstructure, sodium dodecyl sulfate polyacrylamide gel electrophoresis (SDS‐PAGE) and differential scanning calorimetry (DSC) are widely applied to observe the arrangement of muscle structure (Kaale & Eikevik, 2013), semiquantitative analysis in the protein patterns (Yin & Park, 2015), and monitor the protein denaturation during thermal processing (Shao et al., 2018). Our previous research has reported the freezing temperature (liquid nitrogen, −80, −30, and −18℃) and storage duration (1, 4, 12, and 24 weeks) on the properties of red swamp crayfish (Shi, Xiong, et al., 2018). Generally, the biochemical and physical quality of frozen crayfish showed a trend of gradual deterioration with the increase of storage duration. However, it also noticed that no significant changes in the hardness for all cryogenic treatments at week 1, which was not consistent with the trend of Ca^2+^‐ATPase activity and sulfhydryl content of crayfish protein. The correlations among these evaluation attributes have not been reported yet.

Therefore, the objective of this research was to investigate the effects of cryogenic condition on the microstructure, protein pattern, and thermal properties of red swamp crayfish frozen for one‐week storage and explore the internal relationships among these attributes by Pearson's correlation analysis.

## 2 MATERIALS AND METHODS

### 2.1 Experimental design and materials

Fresh alive red swamp crayfish (*Procambarus* *clarkia)* was purchased on May from Qiyimen fresh market, Wuhan. Once arrival to the laboratory, crayfish with the uniform size (9.8 ± 0.5cm) was picked out and cleaned with ice water and then vacuum packed individually. Each ten crayfish (random picked) were set as one batch and subjected to different cryogenic processing for one week. For further analysis, frozen samples were first thawing at 4℃ for 12h and then equilibrating to room temperature.

Three treatments were set 1) −80/‐18, frozen in a −80℃ freezer (DW‐HL340, Zhongke Meling Co., Ltd, China) for 12h and stored at −18℃ (BCD‐215DC, Qindao Haier Co., Ltd, China); 2) −18/‐18, frozen and stored in a −18℃ freezer (BCD‐215DC, Qindao Haier Co., Ltd, China); and 3) −80/‐80, frozen and stored in a −80℃ freezer (DW‐HL340, Zhongke Meling Co., Ltd, China). Fresh samples were set as the control.

### 2.2 Histology

Sample preparation of micromorphology observation was performed according to the method of Jiang et al., (2019). Crayfish tail muscle was dehydration, paraffin embedding, sectioning (cross cut) and dyeing treatment, and then observed by the optical microscope (Eclipse Ci, Nikon, Tokyo, Japan). The magnification of scanned images was adjusted to 1,500 times, and images were collected using Pannoramic Viewer (1.15.3, 3DHISTECH Ltd, Budapest, Hungary). The increase of white regions in the images refers to the damage of cell structure. The number and sum area of white regions, namely WN and WA, respectively, were recorded by Image‐Pro Plus 6.0 (Media Cybernetics, Inc, Rockville, MD, USA) according to the method describe by (Shi, Tao, et al., 2018).

### 2.3 SDS‐PAGE

The protein pattern of crayfish was revealed using SDS‐PAGE according to Jiang et al., (2018) with minor modifications. Ground crayfish tail muscle was homogenized (HBM‐400B, IKA, Germany) with 10‐fold SDS (5%) at 8,000 rpm for 1min. Then, homogenate was water bathed at 90℃ for 1 hr, followed by centrifugation (GL‐21 M, Xiangyi Inc., Changsha, China) at 4,000 rpm for 10 min to obtain the supernatant protein solution. After adjusted the concentration to 1.5 mg/ml, the protein sample was mixed with a loading buffer (1MTris‐HCl (pH 6.8), 50% glycerol, 10% SDS, 14.4 mM β‐mercaptoethanol, 1% bromophenol blue and ddH_2_O) in the ratio of 1:2, following by heating at 90℃ for 5 min. Stacking and separating gels were made using 5% and 10% acrylamide solutions, respectively. Aliquots of samples (7 μl) were loaded onto the gel and subjected to electrophoresis at 120 V. After running, gel was stained with 0.125% Coomassie brilliant blue R‐250 and destained in a solution containing 50% methanol and 10% acetic acid. Gel image was taken using a Gel Doc XR scanner (Bio‐Rad Laboratories, Milan, Italy). The molecular weight of protein bands was determined by comparing with the protein standard (Tiangen Biotech (Beijing) Co., Ltd., Beijing, China). The intensity of protein bands was analyzed by Image J 1.52a (National Institute of Health, Wayne Rasband, USA) according to a method described by Zhu et al., (2017).

### 2.4 Thermal properties

Thermal properties of crayfish tail muscle were measured by differential scanning calorimetry (DSC) using a calorimeter (200F3, Netzsch, Germany). The DSC analyzer was calibrated using indium, and an empty aluminum pan was used as a reference. The samples (about 19~23mg) were prepared in aluminum pans and sealed hermetically, and heated from 10℃ to 90℃ at a rate of 5℃/min. Thermal transitions of Tp (peak of denaturation temperature) and enthalpy of denaturation (ΔH) were calculated and recorded by Proteus software (Netzsch, Germany).

### 2.5 Statistical analysis

GraphPad Prism (GraphPad Software Inc, La Jolla, CA, USA) was performed for graph drawing. ANOVA and Duncan's multiple range tests were used to estimate the statistical significance with a level of *p* <.05 using SPSS (IBM Cop., Armonk, New York, United States). DPS (Zhejiang University, Hangzhou, China) was applied for Pearson's correlation coefficients calculation and correlation analysis.

## 3 RESULTS AND DISCUSSION

### 3.1 Microstructure

**FIGURE 1:**
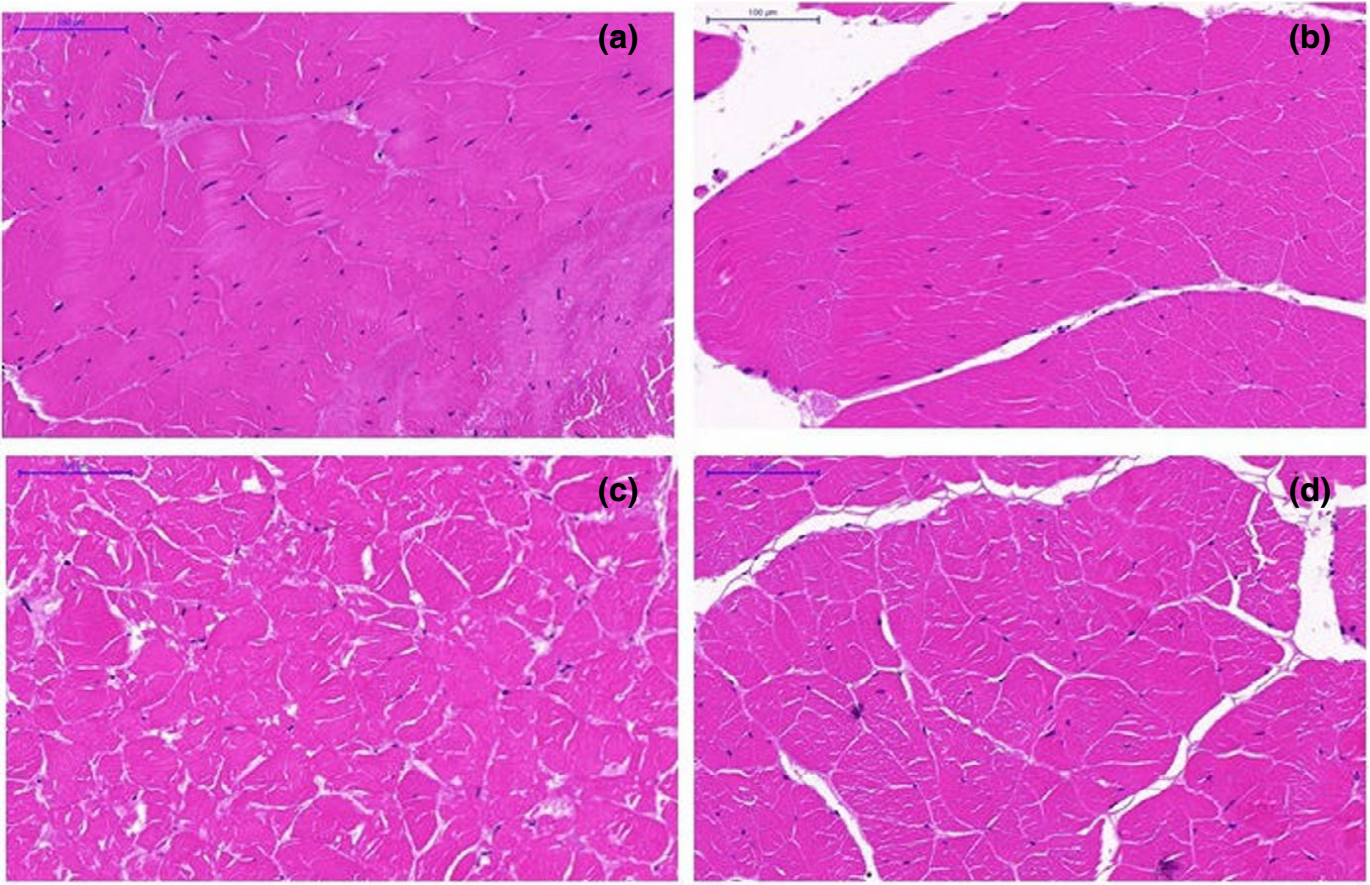
Histology of red swamp crayfish muscle subjected to different cryogenic conditions after one‐week frozen storage. (a) fresh crayfish tail muscle (control); (b) −80/‐80, samples subjected to freezing and stored at −80℃; (c) −80/‐18, samples subjected to frozen at −80℃ and stored at −18℃; (d) −18/‐18, samples subjected to frozen and stored at −18℃. The magnification of scanned images was 1,500 times

Figure 1 depicted the histology of frozen red swamp crayfish muscle subjected to different cryogenic conditions for short‐term storage. Figure 1a displayed a uniform and compact cell structure of fresh crayfish sample (control). Figure 1b (−80/‐80) showed a similar microstructure to the control with relative thicker exomysium. The cell integrity was partially damaged comparing to Figure 1b, and exomysium thickness and cell space was expanded when samples subjected to −18/‐18 treatment (Figure 1d), while the cell units of sample subjected to −80/‐18 treatment presented as completely disordered state (Figure 1c) comparing to other treatments, meaning the severe damage of crayfish tissue. Furthermore, the software Image Pro Plus was applied to analyze the histologic images for digital data. The white regions in the image were seen as the gaps among the tissue cells. Generally, the more and larger the white regions, the higher level of cell damage. According to Table 1, the number and sum area of white region (WN and WA, respectively) in ascending order were both control, −80/‐80, −18/‐18, and −80/‐18. Comparing with the control, WN in the images were increased by 2.82, 3.35, and 5.76 times, respectively. There was no significant difference of WN between the −18/‐18 and −80/‐80 treatment (*p* >.05). While significant difference of WA was noticed among all cryogenic treatment (*p* <.05), WA of −80/‐80, −18/‐18, and −80/‐18 treatment were increased by 1.85, 3.63, and 5.54 times, respectively, comparing with the control.

**TABLE 1:** The number and sum area of white areas in the microstructure image and the intensity of main bands in the SDS‐PAGE image

	Microstructure		SDS‐PAGE					
	WN	WA, μm^2^	MHC	AC	TM	TN T	MLC1	B < 20
Con	9.33 ^a^	116.72 ^a^	109,738 ^a^	109,732 ^a^	78,515 ^a^	102,429 ^c^	88,448 ^b^	87,446 ^b^
−80/−18	53.75 ^c^	646.15 ^d^	97,107 ^a^	101,323 ^a^	79,268 ^a^	64,864 ^a^	63,066 ^a^	58,483 ^a^
−18/−18	31.25 ^b^	424.29 ^c^	122,458 ^b^	120,943 ^b^	87,564 ^b^	86,760 ^b^	69,778 ^a^	66,493 ^a^
−80/−80	26.33 ^b^	216.43 ^b^	101,201 ^a^	119,568 ^b^	93,247 ^b^	85,873 ^b^	95,721 ^b^	97,872 ^b^

Note

Different letters in the same column mean significant difference (*p* <.05).

Abbreviations: AC, actin; B < 20, protein bands with weight molecular lower than 20 kDa; MHC, myosin heavy chain; MLC 1, myosin light chain 1; TM, tropomyosin; TN T, troponin T; WA, sum area of white areas; WN, number of white areas.

**FIGURE 2:**
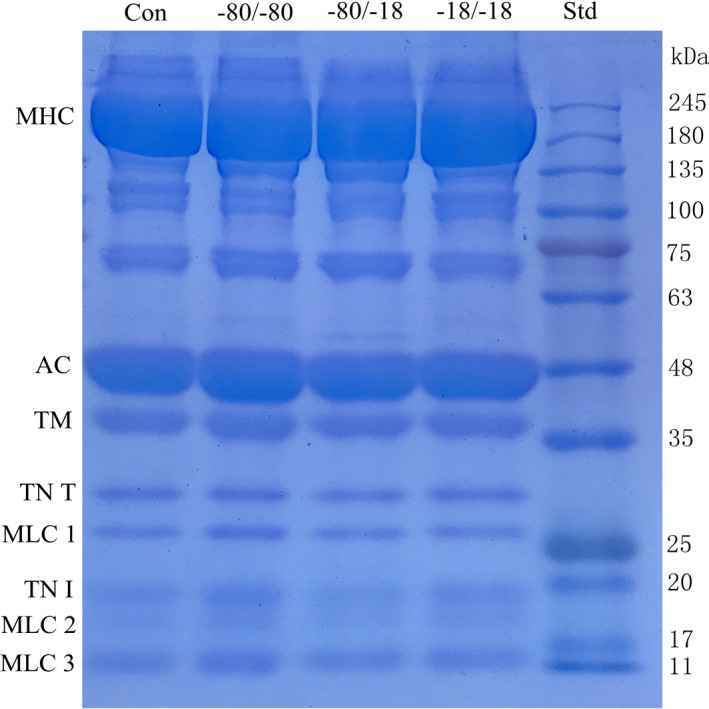
Protein patterns of red swamp crayfish subjected to different cryogenic conditions. Note: Std: protein standard; −80/‐18, samples subjected to frozen at −80℃ and stored at −18℃; −18/‐18, samples subjected to frozen and stored at −18℃; and −80/‐80, samples subjected to freezing and stored at −80℃

The quality of frozen food material was influenced by several processing factors, such as freezing rate (Kaale & Eikevik, 2013), storage temperature, and duration (Lee & Park, 2016). In this research, the crayfish was frozen stored for one week, in the purpose of maximum maintaining the freshness of samples. However, the histomorphology of crayfish muscle was still negative impacted by the cryogenic processing, which indicated the quality deterioration of frozen crayfish. Kaale and Eikevik (2013) believed fast freezing rate was better for food perseveration, since the small and uniform ice crystals were formed during the fast freezing processing without harm the muscle tissue. In this research, WA and WN of −80/‐80 group (freezing rate of 0.325℃ /min) was lower than those of −18/‐18 group (freezing rate of 0.038℃ /min), which proved the Kaale's opinion. Besides, the stability of storage temperature greatly affected the sample quality (Romotowska et al., 2017), which was also confirmed by the high increase rate of WA and WN of the −80/‐18 group (Table 1). The −80/‐18 treatment exhibited a disordered muscle structure (Figure 2c), which might be contributed by the warming effect during frozen storage (Shi, Tao, et al., 2018) and the magnifying effect due to the vulnerable short tissue fiber of crayfish. Comparatively speaking, −80/‐80 treatment was appropriate for crayfish preservation while −80/‐18 was not.

### 3.2 SDS‐PAGE

The protein pattern and band intensities of crayfish subjected to different cryogenic treatments was shown in Figure 2 and Table 1. It was observed that the bands represented MHC (myosin heavy chain, around 220 kDa), AC (actin, 42 kDa), TM (tropomyosin, 40 kDa), TN T (troponin T, 27 kDa), TN I (troponin I, 19 kDa), and MLCs (myosin light chains, 10 ~ 26 kDa) were present in the treated samples. The intensity of main bands in the SDS‐PAGE image was analyzed by Image J. According to Table 1, all band (except TM) of −80/‐18 protein sample had lower intensity than those of other treatments, which suggested the denaturation caused by temperature fluctuation might contribute to protein less extractable (Kjærsgård et al., 2006), while higher intensity of MHC and AC was obtained by −18/‐18 protein sample, and that of TM, MLC 1, and B < 20 (bands with weight molecular less than 20 kDa) was obtained by −80/‐80 protein sample, implying the maintaining of protein structure of crayfish under the same freezing and storage temperature. However, there was no significant difference of the band intensity of MLCs and TN I between −80/‐18 and −18/‐18 sample (*p* >.05), which might be due to protein aggregation attributed by slow freezing rate thus reduced the proportion of small protein (Choi et al., 2017).

### 3.3 DSC

**FIGURE 3:**
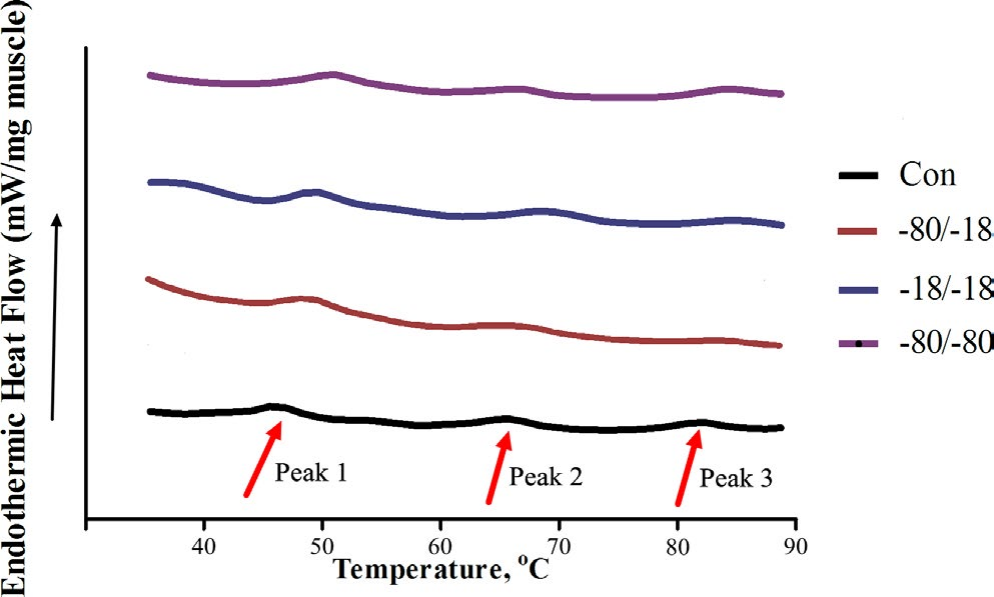
Differential scanning calorimetry (DSC) curve of red swamp crayfish muscle subjected to different cryogenic conditions for one‐week storage. Note: Con: fresh sample; −80/‐18, samples subjected to frozen at −80℃ and stored at −18℃; −18/‐18, samples subjected to frozen and stored at −18℃; and −80/‐80, samples subjected to freezing and stored at −80℃

Figure 3 and Table 2 displayed the thermal properties red swamp crayfish subjected to different cryogenic treatments. Figure 3 exhibited three thermal denaturation peaks between 40 and 90℃, referring to head of myosin (Peak 1), rod tail of myosin (Peak 2), and actin (Peak 3), respectively (Korzeniowska et al., 2013). It was noticed that the position and attitude of three peaks for different treatment were slight moved. As shown in Table 2, Tp (peak of denaturation temperature) of crayfish muscle was significantly raised (*p* <.05) after frozen stored for one week, except for the Tp2 of −80/‐18 treatment. However, no obvious change of Tp was observed among the cryogenic treatments (except Tp1 of −80/‐18 treatment), which suggested it was a moderate denaturation of myofibrillar protein contributed by short‐term frozen storage.

**TABLE 2:** Effects of cryogenic condition on thermal properties of red swamp crayfish

	Peak 1		Peak 2		Peak 3	
	Tp 1(^o^C)	ΔH 1(J/g)	Tp 2 (^o^C)	ΔH 2(J/g)	Tp 3 (^o^C)	ΔH 3(J/g)
Con	47.33 ± 0.21^c^	0.72 ± 0.08^a^	66.53 ± 0.15^a^	0.47 ± 0.08^b^	82.40 ± 0.44^b^	0.33 ± 0.06^a^
−80/−18	48.80 ± 0.35^b^	0.61 ± 0.19^b^	65.97 ± 0.35^b^	0.52 ± 0.08^a^	84.47 ± 0.71^a^	0.15 ± 0.09^c^
−18/−18	49.80 ± 0.99^a^	0.42 ± 0.06^c^	66.70 ± 1.41^a^	0.32 ± 0.13^c^	84.35 ± 0.78^a^	0.16 ± 0.07^c^
−80/−80	49.63 ± 0.55^a^	0.64 ± 0.04^b^	66.07 ± 0.55^ab^	0.54 ± 0.15^a^	84.45 ± 0.49^a^	0.19 ± 0.07^bc^

Note:

Different letters in the same column mean significant difference (*p* <.05).

The value of ΔH (enthalpy of denaturation) revealed the energy required for protein denaturation. It is clearly observed that the ΔH of −18/‐18 treatment was significantly lower than samples freezing at −80℃ (*p* <.05), suggesting that the change of protein conformation was easily happened when freezing under a slow rate. Big and inhomogeneous ice crystals were formed accompanied by slow rate freezing (Kaale et al., 2013), which could lead to cell damage and cytosol loss and resulted in quality deterioration of frozen crayfish product. While on the condition of fast freezing rate, the ΔH of three peaks of −80/‐80 treatment were slightly higher than −80/‐18 treatment (*p* >.05), indicating the storage at lower temperature was benefit for maintaining the stability of muscle protein product.

### 3.4 Pearson's correlation analysis

The Pearson correlation coefficients among the protein band intensity (MHC, AC, TM, TN T, MLC1, and B < 20), microstructure (WN and WA), protein denaturation temperature (Tp1, Tp2, and Tp3), and enthalpy (ΔH1, ΔH2, and ΔH3) are summarized in Table 3. MHC intensity was greatly related to the thermal denaturation of myosin tail, which was that MHC intensity was positively correlated with Tp2 (r = 0.96, *p* <.05), but negatively correlated with ΔH2 (r = −0.96, *p* <.05). Besides, WN was significant positively correlated (*p* <.05) with WA, and negatively correlated (*p* <.01) with the band intensity of TN T. With no doubt, the white regions would increase with the damage of the cell structure. While TN is a regular protein responsible for muscle contraction, the breakage of TN might possibly lead to the increase of cell detachment, namely WN and WA in present work. Therefore, TN T intensity could be potentially applied to reflect the tissue integrity of muscle. Furthermore, MLC 1 showed marvelous correlation (r = 1, *p* <.01) with B < 20 (including TN I, MLC 2, and 3). But, the bands of protein with lower molecular weight were weak and blurry; thus, the relative plain MLC 1 could be considered as the valid label to protein degradation instead.

**TABLE 3:** Pearson's correlation coefficients for structure and physicochemical properties of red swamp crayfish

	WA	WN	MHC	AC	TM	TN T	MLC 1	B < 20	Tp1	Tp2	Tp3	ΔH1	ΔH2	ΔH3
WA	1	0.96*	−0.21	−0.46	−0.22	−0.92	−0.91	−0.89	0.39	−0.37	0.67	−0.49	−0.08	−0.79
WN		1	−0.41	−0.44	−0.07	−0.99**	−0.77	−0.75	0.46	−0.58	0.77	−0.37	0.14	−0.83
MHC			1	0.62	0.13	0.52	−0.11	−0.12	0.17	0.96*	−0.18	−0.65	−0.96*	0.04
AC				1	0.84	0.47	0.45	0.47	0.59	0.49	0.19	−0.48	−0.48	−0.13
TM					1	0.04	0.45	0.48	0.8	−0.06	0.57	−0.36	−0.05	−0.42
TN T						1	0.68	0.66	−0.44	0.68	−0.78	0.27	−0.26	0.80
MLC 1							1	1.00**	−0.18	−0.01	−0.39	0.56	0.39	0.59
B < 20								1	−0.14	−0.03	−0.35	0.55	0.39	0.55
Tp1									1	−0.11	0.91	−0.75	−0.27	−0.87
Tp2										1	−0.45	−0.42	−0.87	0.31
Tp3											1	−0.6	0	−0.97*
ΔH1												1	0.80	0.73
ΔH2													1	0.17
ΔH3														1

Abbreviations: AC, actin; B < 20, protein bands with weight molecular lower than 20 kDa; MHC, myosin heavy chain; MLC 1, myosin light chain 1; TM, tropomyosin; TN T, troponin T; Tp1, Tp2, and Tp3, denaturation temperature of myosin, sarcoplasmic protein, and actin, respectively; WA, sum area of white regions; WN, number of white regions; ΔH1, ΔH2, and ΔH3, denaturation enthalpy of myosin, sarcoplasmic protein, and actin, respectively.

* *p* <.05;

** *p* <.01.

## 4 CONCLUSIONS

This study attempted to explore the internal relationships among tissue microstructure, protein pattern, and thermal properties of red swamp crayfish subjected to different cryogenic treatments. After cryogenic treatments for short‐term storage, cell structure was partially damaged, and protein aggregation and denaturation were presented. Furthermore, WN was negatively correlated (*p* <.01) with the band intensity of TN T, and MLC 1 band intensity was positively correlated (*p* <.01) with the intensity of small proteins (including TN I, MLC 2, and 3). Therefore, this study suggested (1) −80/‐80 treatment was appropriate for crayfish preservation while −80/‐18 was not; (2) the intensive TN T band might suggest the tissue integrity of muscle; and (3) the intensive MLC 1 band could be considered as the valid label to protein degradation.

## CONFLICT OF INTEREST

The authors declared that there are no conflicts of interest regarding the publication of this paper.

## Data Availability

The data that support the findings of this study are available on request from the corresponding author.
